# Antiviral Activity of an Indole-Type Compound Derived from Natural Products, Identified by Virtual Screening by Interaction on Dengue Virus NS5 Protein

**DOI:** 10.3390/v15071563

**Published:** 2023-07-17

**Authors:** Leidy Lorena García-Ariza, Natalia González-Rivillas, Cindy Johanna Díaz-Aguirre, Cristian Rocha-Roa, Leonardo Padilla-Sanabria, Jhon Carlos Castaño-Osorio

**Affiliations:** 1 Grupo de Inmunología Molecular GYMOL, Universidad del Quindío, Armenia 630001, Quindío, Colombia; ngonzalezr@uqvirtual.edu.co (N.G.-R.); cjdiaza@uqvirtual.edu.co (C.J.D.-A.); jhoncarlos@uniquindio.edu.co (J.C.C.-O.); 2 Grupo de Parasitología Molecular GEPAMOL, Universidad del Quindío, Armenia 630001, Quindío, Colombia

**Keywords:** Dengue virus, natural compounds, NS5 protein, antiviral activity

## Abstract

Dengue is an acute febrile illness caused by the Dengue virus (DENV), with a high number of cases worldwide. There is no available treatment that directly affects the virus or the viral cycle. The objective of this study was to identify a compound derived from natural products that interacts with the NS5 protein of the dengue virus through virtual screening and evaluate its *in vitro* antiviral effect on DENV-2. Molecular docking was performed on NS5 using AutoDock Vina software, and compounds with physicochemical and pharmacological properties of interest were selected. The preliminary antiviral effect was evaluated by the expression of the NS1 protein. The effect on viral genome replication and/or translation was determined by NS5 production using DENV-2 Huh-7 replicon through ELISA and viral RNA quantification using RT-qPCR. The *in silico* strategy proved effective in finding a compound (M78) with an indole-like structure and with an effect on the replication cycle of DENV-2. Treatment at 50 µM reduced the expression of the NS5 protein by 70% and decreased viral RNA by 1.7 times. M78 is involved in the replication and/or translation of the viral genome.

## 1. Introduction

Dengue virus (DENV) is a flavivirus transmitted by the bite of female mosquitoes of the genus Aedes spp., endemic in tropical and subtropical countries worldwide [1]. It is the causative agent of the infection known as Dengue or break bone fever [2]. Approximately 400 million cases [3] and 22,000 deaths occur worldwide each year due to Dengue [4]. According to the World Health Organization (WHO), the global incidence of Dengue has dramatically increased in the last decade, and approximately half of the world’s population is at risk [5].

DENV has four genetically distinct serotypes (DENV 1–4). It is an enveloped virus with a single-stranded positive-sense RNA genome that encodes three structural proteins (capsid [C], pre-membrane [prM], and envelope [E]) and seven non-structural proteins (NS1, NS2A, NS2B, NS3, NS4A, NS4B, and NS5) [4,6], each of which perform different functions during the virus infectious cycle. The non-structural proteins are responsible for viral replication and host immune evasion. The NS5 protein plays an essential role in viral RNA replication, as the deletion of this protein from the viral genome inhibits replication [7], making it a promising pharmacological target [8,9]. This protein has two domains, the RNA-dependent RNA polymerase (RdRp) domain at the C-terminal end and the methyltransferase (Mtase) domain at the N-terminal end. The latter is responsible for protecting the RNA at the 5′ end of new viral genomes [1]. 

Despite the significant economic and social impact of this disease and the important advances made against Dengue, there is currently no effective antiviral therapy available [10,11,12]. Considering these limitations, it has become increasingly important to continue the search for molecules, compounds, or drugs that can inhibit enzymatic targets or essential processes for the replication cycle of the virus. The development and search for therapeutic molecules, such as direct-acting antivirals (DAA), has been shown to be a truly effective approach [2]. As a result, the use of computational techniques that have been employed in other research is considered a strategy of interest. Although DAA have not been approved for use in treatment of DENV [13], these have shown great promise in *in vitro* assays. Furthermore, bioactive agents from natural resources have laid a great foundation for the design of new therapeutic drugs [14], allowing a return to the use of traditional medicine to search for treatments for emerging diseases. Similarly, the innovation in the X-ray structures of several DENV proteins has allowed the development of *in silico* computational screening strategies [11]. Therefore, the execution of screenings from databases and docking analysis is promising when selecting an action target, such as important proteins in the virus’s infectious cycle [15,16,17,18,19], with the viral polymerase NS5 protein standing out among these [7]. 

In this research, compounds derived from natural products were identified through virtual screening with an interaction on the NS5 protein. The *in vitro* antiviral effect of an indole-type compound, identified here as M78, was evaluated in a DENV-2 infected cellular model. This evaluation showed that the action is related to intervention during stages of replication and/or translation of the genome.

## 2. Materials and Methods

### 2.1. In Silico Assays

#### 2.1.1. Virtual Screening of Natural Compound Derivatives on DENV NS5 Protein

The structures of NS5 proteins from the four DENV serotypes were obtained as described by García et al. [20]. The selected cavities for interaction corresponded to the substrate binding site of its natural substrate, S-adenosyl homocysteine (SAH), located in the methyltransferase (MTase) domain, and the entrance to the RNA tunnel, present in the RNA-dependent RNA polymerase (RdRp) domain. The ligands SAH and 68E, crystalized in the selected cavities, respectively [21], were subjected to re-docking onto their binding sites to find the coordinates and dimensions of the interaction boxes and obtain a theoretical value of the binding energy as a starting reference point for the selection of the best natural compounds with stronger binding on each region. The Root Mean Square Deviation (RMSD) was calculated as the most commonly used quantitative measure of similarity between two superimposed atomic coordinates [22]. In total, eight virtual screenings were performed (two per serotype). To perform this, the library of 190,090 natural compound derivatives available on the DrugDiscovery@TACC web portal (https://drugdiscovery.tacc.utexas.edu/#/) (accessed on 20 March 2019) [23] from the Texas Advanced Computing Center (TACC) was used. It is worth mentioning that all virtual screening calculations were executed using the AutoDock Vina 1.1 software [24], which is implemented in the DrugDiscovery@TACC web portal.

#### 2.1.2. Selection of Compounds by Interaction on NS5 of DENV

The compounds were selected based on their ability to bind to NS5 in the four serotypes of DENV. Predictions of aqueous solubility were performed using the SwissADME web server (http://www.swissadme.ch/) [25]. This server delivers three predictions for this physicochemical descriptor with six possible outcomes: insoluble, poorly soluble, moderately soluble, soluble, highly soluble, and very highly soluble. We used a score of 0 for the descriptors of insoluble and poorly soluble, a score of 1 for the descriptors of moderately soluble and soluble, and a score of 2 for the descriptors of highly soluble and very highly soluble. Thus, only compounds that obtained a value of two or higher (by summing the scores of the three predictions provided by the SwissADME server) were selected for the next filter. A prediction was made of compliance or violation of the four Lipinski rules (Molecular weight ≤ 500, LogP ≤ 5, hydrogen bond acceptors ≤ 10, hydrogen bond donors ≤ 5) [26]; thus, only compounds that had a maximum of one violation were accepted for the next filter. This prediction was made using the SwissADME server. Prediction of possible toxicological risks using the ProTox-II web server (http://tox.charite.de/protox_II/) [27], such as hepatotoxicity, carcinogenicity, immunotoxicity, mutagenicity, and cytotoxicity, were carried out, and only those compounds that did not present toxicological risks after the prediction were selected. 3D visualizations of protein-ligand complexes were performed with the Chimera v1.13.1 program [28].

### 2.2. In Vitro Assays

#### 2.2.1. Determination of the Cytotoxic Effect of Compounds on Huh-7 Cells

The compounds identified and selected through *in silico* assays were acquired through a synthesis service at MolPort (https://www.molport.com/shop/index). Subsequently, the cytotoxic effect on Huh-7 cells (ATCC HB 8065) was evaluated using the cell viability assay, using resazurin as a metabolic indicator. For this purpose, 15,000 cells were cultured per well in DMEM medium (Dulbecco’s Modified Eagle Medium, Life Technologies 12100-046, New York, NY, USA), supplemented with 10,000 units/mL of penicillin/streptomycin, 20 mM L-glutamine, and 2% (*v/v*) heat-inactivated fetal bovine serum (Eurobio, CVFSVF00-01, Les Ulis, France) in 96-well Multiwell plates (Costar 3590, New York, NY, USA). The cell monolayer was allowed to stabilize for approximately 24 h at 37 °C and 5% CO_2_. Subsequently, the cells were treated with the compounds at concentrations of 12.5, 25, 50, 100, 125, 250, and 500 µM. Cell viability was determined after 24 and 48 h of compound exposure, using resazurin at a final concentration of 44 µM, incubating for 2 h under the previously described conditions. Finally, absorbance was measured at 603 and 570 nm. The percentage of cell viability was calculated considering the difference between the absorbances for each treatment and the untreated cell control (CC), using the following formula:

Cell viability (%) = [Sample absorbance/Control absorbance] × 100 (1)

The mean cytotoxic concentration (CC_50_) was also established, defined as the concentration at which cell viability decreases by 50%.

#### 2.2.2. Antiviral Screening of Compounds on NS1 Protein Production in DENV-2 Infected Cells

For antiviral screening, 15,000 Huh-7 cells (ATCC HB 8065) were cultured per well in 96-well Multiwell plates (Costar 3590, New York, NY, USA) under the same conditions as described in item 2.2.1, and then these were infected with DENV-2 New Guinea for 2 h at a multiplicity of infection (MOI) of 1. The DENV-2 strain used was isolated and cultivated in C6/36 mosquito cells (ATCC^®^ CRL-1660) and maintained in L-15 medium supplemented with 10% tryptose and 2% fetal bovine serum, incubated for seven days at 28 °C and stored at −80 °C. This strain was provided by the Biomedical Research Center of the University of Quindío, Colombia. After infection, the supernatant was removed, and the compounds were added at non-cytotoxic concentrations (between 40 and 100 µM, previously determined) and incubated for 24 h. Mycophenolic acid 20 μM [29] was used as an inhibition control, and 0.45% DMSO (vehicle) was used as a negative control. Subsequently, the cells were treated with 4% paraformaldehyde for 30 min and permeabilized for 5 min with 1X PBS, 0.5% Triton. The cells were then blocked with 5% fetal bovine serum in 0.05% PBS-Tween for 24 h at 4 °C. The primary Monoclonal anti-Dengue Virus NS1 antibody (SAB2702307 Sigma Aldrich, Saint Louis, MO, USA) (1:1000) was added and incubated for 1 h and 30 min at 37 °C. The secondary antibody, Anti-Mouse IgG (whole molecule) −Alkaline Phosphatase antibody produced in goats (A3562-Sigma Aldrich), was then added and incubated for 1 h at 37 °C. Finally, the alkaline phosphatase substrate pNPP (S0942-Sigma Aldrich) was added and incubated for 30 min. Then, NaOH 0.1 M solution was added, and absorbance was measured at 405 nm on an Epoch spectrophotometer. Absorbance values were transformed into percentages of NS1 production and compared to the viral control (VC), using the following formula:

NS1 production (%) = [Sample absorbance/Control absorbance] × 100(2)

#### 2.2.3. Determination of the Inhibitory Effect on NS5 Protein on the Expression Using ELISA Technique

To determine the effect of the compound on protein expression, the production of NS5 was evaluated using the Huh-7 cell line, which carries a DENV-2 subgenomic replicon. The replicon includes a luciferase reporter gene, a geneticin resistance gene, and the coding region of NS proteins (NS1 to NS5), allowing for stable expression of these proteins. These systems contain genetic elements necessary for autonomous genome replication in cells and have been useful for expressing viral genes in several flaviviruses, including DENV, WNV, YFV, and TBEV [30]. The cells were cultured in DMEM (Dulbecco’s Modified Eagle Medium, Life Technologies 12100-046, New York, NY, USA), supplemented with 10,000 units/mL of penicillin/streptomycin, 20 mM L-glutamine, and 10% (*v/v*) heat-inactivated fetal bovine serum. Geneticin G418 (10131-035, Gibco, Grand Island, New York, NY, USA) was added at a final concentration of 0.2 mg/mL as a selection antibiotic for the transfected cells with the replicon. This cell line was provided by the Biomedical Research Center of the University of Quindío, Colombia. To begin, 15,000 Huh-7 DENV-2 replicon cells were cultured per well in 96-well Multiwell plates (Costar 3590, New York, NY, USA). After reaching 70–80% confluency, they were treated with compounds that had previously shown an effect on the viral cycle and were incubated for 24 h at 37 °C and 5% CO_2_. NITD008 compound, an NS5 protein inhibitor [31], was used as a positive control, along with other respective controls. The cells were treated with 4% paraformaldehyde for 30 min, and permeabilized for 5 min with a 0.5% PBS 1X Triton X100 solution. Then, the cells were blocked with 5% fetal bovine serum in 0.05% PBS-Tween for 24 h at 4 °C. Then, the cells were treated with primary Anti-NS5 antibody produced in rabbits (SAB2700025 Sigma Aldrich) (1:10,000) and incubated for 1 h and 30 min at 37 °C. Then, goat anti-rabbit (whole molecule) alkaline phosphatase-conjugated antibody (A3687-Sigma Aldrich) diluted 1:30,000 was added and incubated for 1 h at 37 °C. Subsequently, alkaline phosphatase substrate (S0942-Sigma Aldrich^®^) was added for 1 h at 37 °C, followed by the addition of 0.1 M NaOH solution, and the absorbance was measured at 405 nm using an Epoch spectrophotometer. The absorbance values were transformed into NS5 production percentage and compared to the viral control, using the (2) formula. The IC_50_ was estimated through the dose-response curve, using the GraphPad Prism 6 software, and the selectivity index (SI) was also predicted by calculating the ratio between CC_50_/IC_50_.

#### 2.2.4. Determination of the Inhibitory Effect on Viral RNA Production of DENV-2

After treatments with the selected compounds on the previously infected Huh-7 cells with DENV-2, as described above, total RNA extraction was performed using the TRIzol LS Reagent^®^ (Lot.50867000) following the manufacturer’s recommendations. The concentration and purity of the RNA were determined by the absorbance ratio at 260 nm and 280 nm, read on an Epoch spectrophotometer. Subsequently, the amplification of the NS5 protein gene was performed using the primers DENV 7764 Fwd 5′-CGTCGAGAGAAATATGGTCACACC-3′ and DENV 7844 Rev 5′-CCACAATAGTATGACCAGCCT-3′. The endogenous GAPDH gene was amplified using the primers hGAPDH Fwd 5′-TGTTGCCATCAATGACCCCTT-3′ and hGAPDH Rev 5′-CTCCACGACGTACTCAGCG-3′. RT-qPCR was performed using the Power SYBR^®^ Green RNA-to-CTTM 1-Step kit (Ref 4389986, Applied Biosystems^TM^), following the manufacturer’s instructions, for a total reaction volume of 20 μL. As a negative amplification control, a reaction mixture without genetic material was included. The RT was performed at 48 °C for 30 min, enzyme activation at 95 °C for 10 min, denaturation for 40 cycles of 95 °C for 15 s, and annealing and extension at 60 °C for 1 min. The relative expression of this gene was calculated using the comparative CT method (2^−ΔΔCT^) [32], which makes several assumptions, including that the PCR efficiency is close to 1 and the PCR efficiency of the target gene is similar to that of the internal control gene, using the following equation:

2^−ΔΔCT^ = [(CT gene of interest − CT internal control) Sample A − (CT gene of interest − CT internal control) Sample B]. (3)

### 2.3. Statistical Analysis

In all cases, treatments were compared with their respective controls. A Shapiro–Wilk test was performed to evaluate data normality. Parametric data were evaluated using one-way ANOVA with multiple comparisons test through Dunne’s t method. Non-parametric data were evaluated using Kruskal–Wallis test, with comparison test through Dunn’s test. *t*-test was performed to compare two groups of parametric data, Tukey test to compare means, and Mann–Whitney U test for two groups of non-parametric data. A *p*-value < 0.05 was considered statistically significant. The analyses were performed using GraphPad Prism 6 software.

## 3. Results

### 3.1. The MTase and RdRp Domains Were Validated As Binding Sites

The crystallized structure of NS5 protein from serotype 3, PDB 5JJR, containing two ligands on the regions of interest of the MTase and RdRp domains (SAH and 68E, respectively), was used to re-dock these two compounds onto the NS5 protein of all DENV serotypes. The defined dimensions for all boxes were 24 Å in all axes (x, y, and z). 

**Figure 1:**
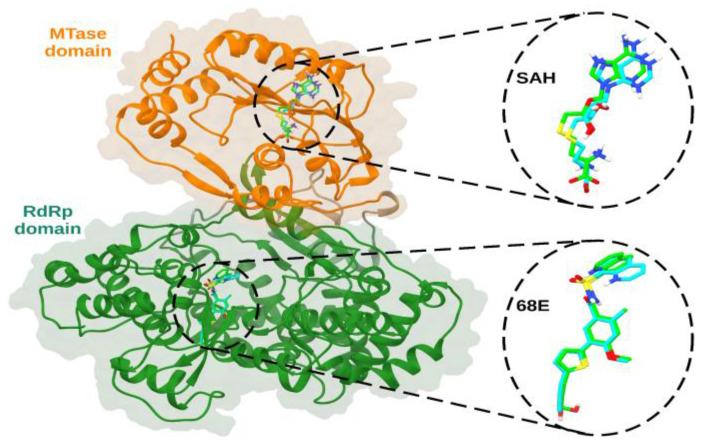
Molecular re-docking of co-crystallized ligands onto NS5 protein of serotype 3 of DENV (PDB 5JJR). SAH ligand in MTase domain, with an RMSD of 1.2 Å, and 68E ligand in RdRp domain, with an RMSD of 1.7 Å. The MTase and RdRp domains are represented in orange and dark green ribbons, respectively. The crystallized and predicted poses are shown in green and cyan sticks, respectively.

In Figure 1, the re-docking of the SAH and 68E ligands onto the NS5 protein of serotype 3 is presented. As shown, molecular docking was able to approximately reproduce the crystallographic pose of the control ligands. In the case of PDB 5JJR, a value of 1.2 Å was obtained for the RMSD between the crystallized SAH ligand and the predicted pose, and for the case of the 68E ligand present in the RdRp domain, a value of 1.7 Å was obtained for the RMSD. The calculated interaction energies for SAH was −7.6 kcal/mol, and for 68E was −8.6 kcal/mol.

### 3.2. Selected Compounds by Interaction on MTase and RdRp of DENV NS5 Protein

**Figure 2:**
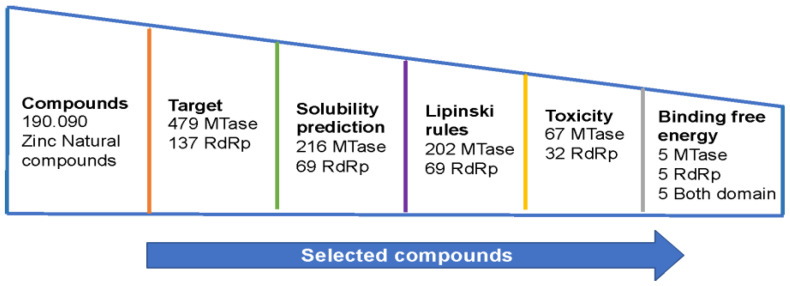
Stages and selection criteria for the selection of natural compounds with potential anti-DENV activity.

Figure 2 shows the steps taken to select compounds from the Zinc Natural Compounds database that have interaction with the DENV NS5 protein.

After selecting the binding sites and setting up the interaction boxes used in the compound search, virtual screening was performed. Initially, 190,090 natural compounds were docked to the two binding sites of interest (MTase and RdRp) in the four serotypes. This means that a total of eight virtual screenings and approximately 1,520,720 molecular dockings were performed on the NS5 protein models of DENV. The DrugDiscovery@TACC web portal [23] provided the top 1000 compounds for each screening, and the list was reduced to 8000 natural compounds (4000 for each domain). In order to identify compounds that may exhibit anti-Dengue effects, only compounds that interacted with the NS5 protein domains in all Dengue serotypes were selected. After verifying the compounds at each binding site, a total of 479 compounds were obtained in the MTase domain and 127 compounds were obtained in the RdRp domain in all four serotypes. Finally, physicochemical and toxicological property screening was performed. The solubility evaluation in aqueous systems allowed for the selection of 216 compounds for the MTase domain and 69 compounds for the RdRp domain. Then, the Lipinski rule compliance was checked, resulting in 202 compounds selected for the MTase domain and all previously identified compounds for the RdRp domain. The next analysis was based on the predictions of possible toxicological risks, which resulted in 67 compounds for the MTase domain and 32 compounds for the RdRp domain. The final selection of compounds was based on the binding energies. Five compounds were chosen for the MTase domain, five for the RdRp domain, and five with interaction in both domains (Table 1). The compounds were named according to the interaction site and the last two digits of the Zinc code. Based on the results, the compounds were acquired from Molport (https://www.molport.com/shop/index) (accessed on 30 August 2019) for synthesis and to begin the *in vitro* evaluation of the molecules’ activity against DENV-2.

### 3.3. Cytotoxic Effect Evaluation of Compounds in Huh-7 Cells

The cytotoxic effect was evaluated for 10 of the 15 identified compounds (M66, M76, M78, R07, R32, R53, R55, MR25, MR41, and MR94). According to the results, among the compounds with interaction on the MTase domain, M66 caused a decrease in cell viability (≥ 50%) at concentrations of 50 and 100 µM, with statistically significant differences when compared to the control cells (**** *p* < 0.0001). The estimated CC_50_ was 44.08 µM. For compound M76, no effect was observed on cell morphology or metabolism at the highest concentration evaluated (100 µM) after 24 h of treatment exposure; therefore, CC_50_ was not determined. On the other hand, after treatment with compound M78, cellular viability close to 100% was evidenced at concentrations of 12.5, 25, and 50 µM, while at 100 µM, damage to the monolayer and therefore loss of viability was observed, with statistically significant difference when compared to the control cells (**** *p* < 0.0001), finding a CC_50_ of 60.77 µM. Regarding the compounds identified with interaction on the RdRp domain, it was found that compounds R07, R32, and R55 did not reduce cell viability at any of the evaluated concentrations. On the other hand, treatment with compound R53 reduced cell viability at all evaluated concentrations, with evident cell death. The determined CC_50_ for the compound was 30 µM. On the other hand, for compounds with binding in both domains, CC_50_ of 70.57 µM was found for MR25 and CC_50_ of 46.22 µM for MR94, and no effect was evidenced for MR41 at any of the evaluated concentrations, so CC_50_ was considered >100 µM (Table 2).

### 3.4. The Compounds Reduce the Production of NS1 Protein in Cells Infected with DENV-2

**Figure 3:**
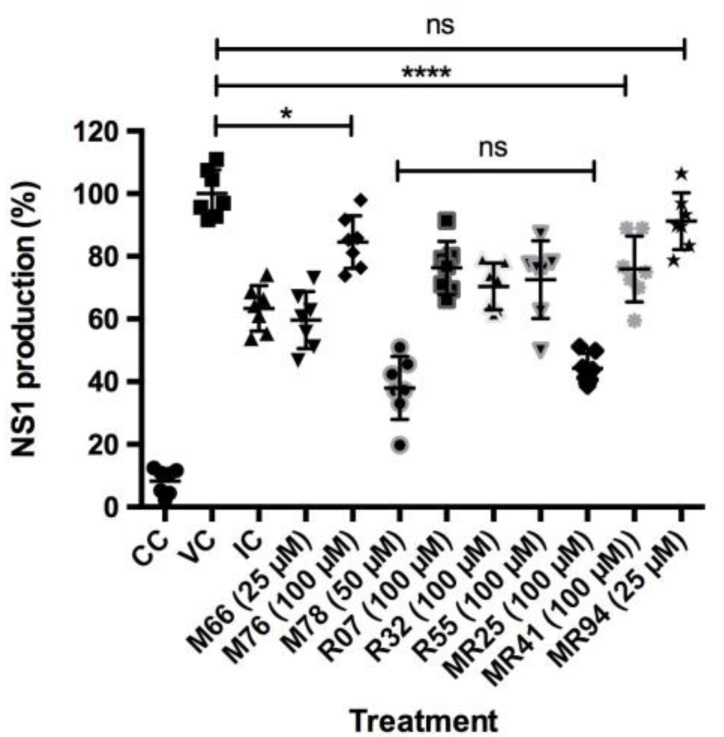
Percentage of NS1 protein production after treatment with compounds M66, M76, M78, R07, R32, R55, MR25, MR41, and MR94 in DENV-2 infected cells, as measured by ELISA. VC: Viral control, CC: Cell control, CI: Inhibition control (20 μM mycophenolic acid). Data are represented as mean and standard deviation (n = 7). One-way ANOVA analysis indicated statistically significant differences between the compounds and viral control (VC) (*** *p* < 0.001), (* *p* < 0.1; **** *p* < 0.0001), except for compound MR94. *t*-test indicated no difference between M78 and MR25 (ns: non-significant).

The antiviral activity of the compounds was determined for nine (9) out of the ten (10) compounds previously evaluated, excluding compound R53, which showed higher toxic effects with a CC_50_ of 30 µM. This first selection analysis was performed through an ELISA assay with detection of the DENV NS1 protein. According to these results, there was a reduction in NS1 production after treatment with eight out of nine compounds (M66, M76, M78, R07, R32, R55, MR25, MR41), with statistically significant differences when compared to the viral control (**** *p* <0.0001), except for treatment with MR94. It is worth noting that the addition of M78 and MR25 reduced the expression of this protein, with production rates close to 38% and 45%, respectively. These rates were lower than that presented by the inhibition control (mycophenolic acid), which was around 63% (Figure 3).

### 3.5. Compound M78 Affects NS5 Protein Production by Interfering with Genome Replication and/or Translation

**Figure 4:**
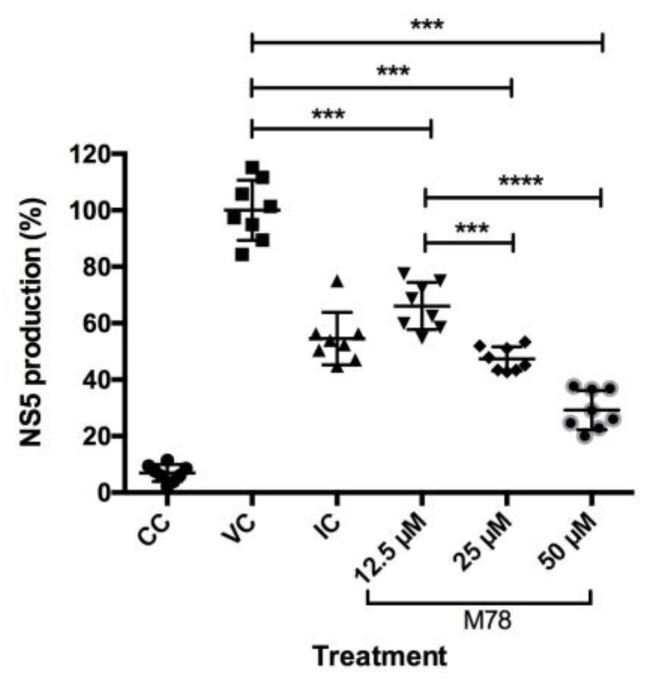
Effect of M78 compound on the production of DENV-2 viral protein NS5 in Huh-7 replicon cells using ELISA assay. CC: Cell control, VC: Viral control, IC: Inhibition control (20 µM Mycophenolic Acid). Data represent mean and standard deviation (n = 8). One-way ANOVA analysis indicates statistically significant differences between all concentrations of M78 evaluated in relation to viral control (*** *p* < 0.001). Tukey test indicates difference between the three M78 treatments, between 12.5 µM and 25 µM (*** *p* < 0.001), and between 12.5 µM and 50 µM (**** *p* < 0.0001).

The compound M78 was selected because it showed the greatest reduction in NS1 protein production when compared to the other compounds, decreasing expression by approximately 60%, according to the previously described results (Figure 3). M78 was evaluated at lower concentrations to assess its effect on NS5 expression. The ELISA results are presented in Figure 4, indicating the percentage of protein production and showing dose-dependent reduction with statistically significant differences when compared to viral control (VC) (*** *p* < 0.001). However, treatment with the compound at three different concentrations reduced protein production to values close to those observed in the positive inhibition control. Nevertheless, there were statistically significant differences between the three treatments, indicating a greater effect of M78 at 50 µM, where NS5 production was close to 30% compared to VC (100%). The IC_50_ was estimated, with a value of 24.61 μM, and according the determined CC_50_, an SI of 2.5 was found.

### 3.6. The M78 Compound Affects The Production of DENV-2 Viral RNA

**Figure 5:**
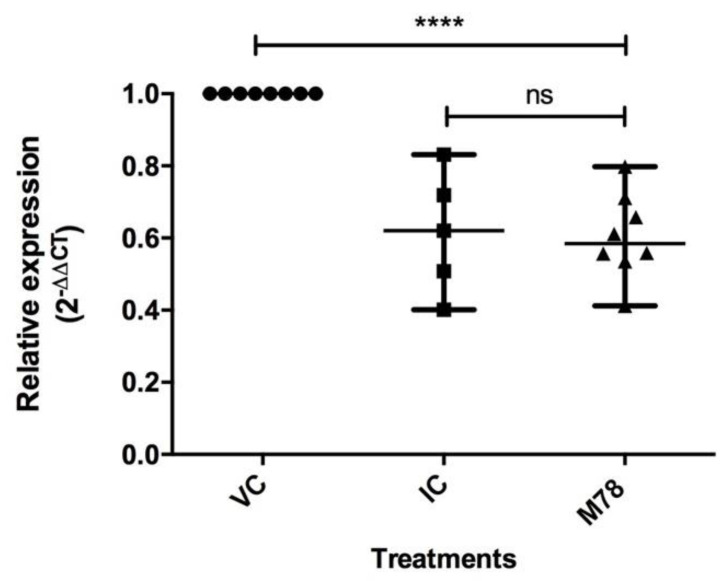
Effect of compound M78 (50 µM) on the production of DENV-2 viral RNA determined through relative expression levels, by RT-qPCR, normalized using GAPDH. VC: Viral control, IC: Inhibition control (20 µM Mycophenolic acid). Data represents the median and interquartile range (n = 8). Kruskal–Wallis test analysis shows statistically significant differences between treatment with compound M78 and inhibition control (IC) compared to viral control (VC) (*** *p* < 0.001). Mann–Whitney test indicates no difference between inhibition control (IC) and M78 (ns: non-significant).

The results of the antiviral effect and protein expression after treatment with 50 µM of M78 led to the evaluation of the compound’s action on viral RNA synthesis. The evaluation showed a decrease in the relative expression of the DENV-2 gene when compared to the viral control (Figure 5).

Figure 5 shows the relative expression levels for VC, IC, and M78 treatment. The results indicate statistically significant difference between treatment with M78 and viral control (**** *p* < 0.0001). Based on this, it is possible to state that the expression of the evaluated gene was reduced 1.7 times due to the compound treatment, with similar behavior to that of the inhibition control.

## 4. Discussion

The use of bioinformatics tools for the study of molecular docking has become an important component in the drug discovery process [33]. Over the past two decades, computational technologies have played a crucial role in the development of antiviral drugs [34]. For DENV, the NS5 protein has been reported as an important target for the search for new molecules with inhibitory capacity of its function, knowing that it does not have homologues in the eukaryotic cell, which decreases the probability of toxic effects. It is highly conserved in flaviviruses, and within this protein, the thumb subdomain in RdRp plays a crucial role in assisting in the synthesis of viral RNA [2]. Likewise, the MTase domain has essential enzymatic activity in replication and positively influences polymerase activity [20,35]. Considering this, the molecules that bind to these sites may hinder conformational changes for RdRp activity [21]. Seeking compounds that bind in these regions remains an objective, since it has been demonstrated that the development of therapeutic molecules, such as direct-acting antivirals (DAA), is a truly effective approach [2].

The NS5 structures of the four serotypes were obtained from García et al. These models include most of the NS5 amino acids from all serotypes, and they are reliable and comparable based on validation of Z-score values against structures resolved by X-ray and NMR and torsion angles [20]. Molecular docking with natural compounds was performed on the SAM and RNA tunnel regions of NS5, after the re-docking of the SAH and 68E ligands on the NS5 protein of serotype 3. The RMSD values obtained between the computationally calculated and experimental poses for the control ligands SAH and 68E were 1.0 Å and 0.8 Å, respectively (Figure 1), presenting small values on an atomic scale. A value equal to 0 indicates identical structures, and as the two structures become different, its value increases [36], suggesting that the two compared structures are very close to having the same formation. In other words, this result supports the interaction coordinates used in the re-docking, indicating that they are suitable in the search for natural compounds with interaction in DENV NS5, known as the most conserved protein, and considered a promising pharmacological target due to its fundamental role in replication, viral RNA methylation, RNA polymerization, and evasion of the host immune system [8].

Molecular docking has become an essential component for drug development and represents an approach that can aid in therapeutic and pharmaceutical development. The purpose of evaluating the antiviral potential of compounds derived from natural products is aimed at their proven success in many pharmacological therapies. As such, and because they present antiviral properties, they may be an alternative target for drug development in order to combat the Dengue virus [37]. The binding energies of compounds on NS5 protein were found to be between −9.2 and −12 kcal/mol (Table 1), indicating the global minimum energy of the complex formed between ligand and receptor [38]. The molecular docking results suggest that all our compounds have a higher binding site affinity than their respective controls, based on re-docking (for SAH, this was −7.6 kcal/mol; for 68E this was −8.6 kcal/mol). This condition was considered as an initial criterion when choosing the compounds. The binding energy is often used to determine the affinity of biomolecular interactions and drug efficacy [39]; the more negative the binding affinity, the stronger the ligand-receptor interaction and the better molecular docking prediction [40]. Further, predictions of physicochemical and toxicological properties favored the selection of these compounds. Compliance with Lipinski’s rules was established as an important criterion for this classification, as higher molecular weight is associated with a lower rate of permeability in lipid bilayer membranes, and a LogP less than five has approximately a 90% probability of being orally soluble. When a drug-like molecule satisfies the five fundamental principles, it exhibits greater pharmacokinetic properties and bioavailability [37], making it feasible to consider whether the compounds possess possible drug properties and can be used in the future as candidates [20,41]. Likewise, predicting possible risks favored ordering the compounds in order to evaluate the most promising ones *in vitro.* This allowed for the postulation of 15 candidate compounds for *in vitro* evaluation in DENV.

The experimental phase began with the determination of the cytotoxicity of 10 of the 15 identified compounds. The results showed that six of them had CC_50_ in Huh-7 cells above 100 µM (M76, R07, R32, R55, MR25 and MR41), while the CC_50_ of the others were less than or equal to that found for M78 (60.77 µM). No apparent cellular damage was found up to 50 µM (Supplementary Figure S1) so it was decided to evaluate these compounds at lower concentrations relative to the CC_50_ determined for each one (Table 2). According to the chemical structure of the compounds, eight out of ten evaluated *in vitro* contain a scaffold of five-ring named 7,8,13,13b-tetrahydro-5H-benz [1,2] indolizino [8,7-b] indole. Indole compounds possess pharmacological potential that has been used as an excellent scaffold in the discovery of antimicrobial drugs, anticancer agents, antihypertensive, antiproliferative, and anti-inflammatory agents [42]. The activity of these compounds is associated with the molecular interactions generated between the indole compound and the therapeutic target [43], with high affinity, which aids in the development of new biologically active compounds [44,45]. Medically useful or promising indole compounds span the entire structural spectrum, from simple indoles to highly complex indole alkaloids [43]. Recent studies have pointed out that these types of structures exhibit an effect against flaviviruses [46] such as DENV, and other viruses such as HIV and influenza virus [47], and that fused tricyclic derivatives of indoline and imidazolidinone have action on ZIKV and DENV infection [48].

The effect of the compounds against DENV was initially evaluated for nine out of the ten selected compounds (excluding R53) by measuring the expression of DENV-2 NS1 protein, which was used as a model because it is one of the most prevalent serotypes [49]. The results indicated that all compounds, except MR94, reduced NS1 expression when compared to the viral control (Figure 3). The presence of NS1 confirms Dengue infection and serves as evidence of successful viral replication [9,50]. Among the compounds, M78 showed better effects by decreasing protein production by over 60% (production close to 38%). In other studies, the detection of DENV-2 NS1 through ELISA also identified that hydroalcoholic extracts of leaves (UGL) and bark (UGB) from the medicinal species *Uncaria guinanensis* reduced the levels of this protein at concentrations of 0.5, 5, and 10 µg/mL [51]. Considering that NS1 is a multifunctional protein essential for virus production, and that, in infected cells, it is necessary for the formation of virus-induced membrane structures that serve as replication sites for DENV [52], the findings presented here support the concept of an antiviral action by the evaluated compounds.

On the other hand, it was found that compound M78 induces a reduction in NS5 expression, with a dose-dependent effect (Figure 4), and a predicted selectivity index (SI) of 2.5 µM, indicating that the compound is effective and selective at this concentration. This parameter is accepted to express the efficacy of a compound in inhibiting viral replication, although studies have shown that SI values < 10 have limited antiviral activity, as observed in the case of OA, a methylated flavone from *Oroxylum indicum*, with an SI of 2.66 against DENV-2 [53]. Furthermore, by using the replicon system, which allows studying aspects of viral replication due to the lack of structural genes [30], it is possible to consider that the intervention of M78 is directed towards viral replication and/or the expression of proteins associated with this process [54], which is also evidenced by the reduction in viral RNA copies (Figure 5), where a 2^−ΔΔCT^ value less than 1 indicates a reduction in gene expression due to the treatment [32], estimating that such reduction was 1.7 times greater compared to the viral control. Based on the above, we can say that M78 intervenes in the viral cycle of DENV-2, although the mechanism through which this effect occurs requires further validation, using complementary techniques that allow for a deeper understanding of the role of this compound as an antiviral.

Other studies have reported the evaluation of compounds and plant extracts against DENV-2; among them, it has been indicated that the ethanolic extract of *A. calamus* root (Tatanan A) presented a similar effect to that found for M78, related to the intervention in the initial stage of viral replication, while inhibiting DENV-2 mRNA and protein levels [55]. On the other hand, it has also been published that hirsutine, an alkaloid from *Uncaria rhynchophylla* that shares structural similarity with M78 in relation to the indole nucleus, was identified as a potent anti-DENV compound in the four serotypes, inhibiting viral particle assembly, budding, or release step, but not translation and viral replication in the DENV lifecycle [56]. However, we have found a different dynamic for the compound M78, demonstrating that the compound intervenes in DENV-2 viral replication by acting on RNA synthesis and/or translation of the viral genome, causing a decrease in the production of viral proteins, as we have observed (Figure 4 and Figure 5). Furthermore, considering that indole is a potent basic pharmacophore present in a wide variety of antiviral agents [45], it is also known that some indole derivatives have been effective and selective inhibitors of this virus replication [57]. In this sense, the design of antiviral drugs containing indole is useful for combating viral infections [42], and furthermore, its application is interesting if this type of compound is found in natural products, as compounds from these sources have prevented DENV from infiltrating the genome or act by reducing structural and non-structural proteins that are produced [58]. This supports our findings. The identification of this naturally occurring compound is interesting, as similar bioactive compounds with antiviral properties could be combined with existing therapies along with different administration methods to enhance their efficacy [59].

Furthermore, our results indicate that the *in silico* strategy used in this research to search for compounds against Dengue proved to be effective, allowing the identification of 15 compounds with interaction in NS5 from DENV-1 to DENV-4 out of a total of 190,090 evaluated natural compounds. Among them, compound M78 showed *in vitro* antiviral activity, highlighting the utility of this methodology for future studies in the identification of compounds targeting viral targets. The findings suggest that the natural compound M78 could be considered a candidate for DENV-2. M78 is involved in viral replication, and it is recommended to study its role in NS5 in more detail, as well as its action in pre-treatment and its virucidal effect against other Dengue serotypes and flaviviruses. It is important to note that its chemical structure, for which no other biological activity assays are currently known, possesses an indole core that could be associated with its antiviral effect, which increases the interest in further investigating this compound identified here through virtual screening.

## Figures and Tables

**Table 1:** Natural compounds identified through virtual screening with interaction in the NS5 protein of DENV.

Binding Site	Zinc Code	Assigned Name	Binding Energy (kcal/mol)			
			DENV-1	DENV-2	DENV-2	DENV-4
Mtase Domain	ZINC08790808	M08	−11.4	−11.4	−11.5	−11.5
	ZINC03839432	M32	−11.1	−11.1	−11.1	−11.2
	ZINC08791166	M66	−11.4	−11.5	−11.5	−11.4
	ZINC35485176	M76	−12.0	−12.1	−12.2	−12.0
	ZINC08790178	M78	−12.0	−12.1	−12.2	−12.0
RdRp Domain	ZINC02094107	R07	−9.8	−9.7	−9.8	−9.6
	ZINC04085432	R32	−10.1	−10	−10.5	−9.9
	ZINC04085246	R46	−9.8	−9.7	−10.1	−9.2
	ZINC12884853	R53	−9.8	−9.8	−10.1	−9.3
	ZINC20611155	R55	−9.8	−9.6	−9.8	−10.0
Both domain	ZINC08790125	MR25	MTase −11.8	−11.9	−12.1	−11.9
			RdRp −10.1	−9.8	−10.0	−9.7
	ZINC08791241	MR41	MTase −11.2	−11.3	−11.4	−11.3
			RdRp −10.0	−9.9	−10.4	−9.4
	ZINC12885588	MR88	MTase −11.2	−11.1	−11.3	−11.2
			RdRp −10.4	−10.3	−10.8	−10.2
	ZINC04086794	MR94	MTase −11.1	−11.2	−11.4	−11.1
			RdRp −10.2	−10.4	−10.4	−10.2
	ZINC08791299	MR99	MTase −11.3	−11.3	−11.5	−11.3
			RdRp −10.2	−10.1	−10.4	−9.9

**Table 2:** Mean cytotoxic concentration (CC_50_) of evaluated compounds on Huh-7 cell line.

Compound	Structure	CC_50_ (µM)
M66	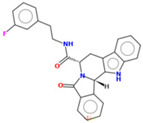	44.08
M76	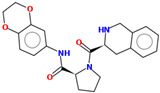	>100
M78	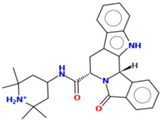	60.77
R07	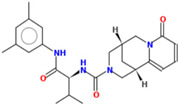	>100
R32	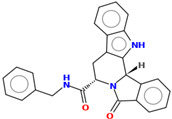	>100
R53	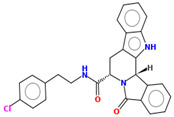	30
R55	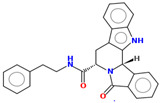	>100
MR25	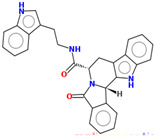	>100
MR41	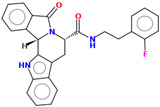	>100
MR94	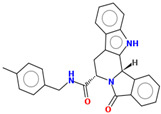	46.22

## Data Availability

The study did not report any data.

## Supplementary Materials

The following supporting information can be downloaded at: https://www.mdpi.com/article/10.3390/v15071563/s1, Figure S1: Cell viability of Huh-7 for compound M78, as measured by resazurin assay.
