# Supplementary figures and images for: Antiviral Activity of an Indole-Type Compound Derived from Natural Products, Identified by Virtual Screening by Interaction on Dengue Virus NS5 Protein
Source: Viruses. 2023 Jul 17;15(7):1563. doi: 10.3390/v15071563 (PMC10384440; doi:10.3390/v15071563)

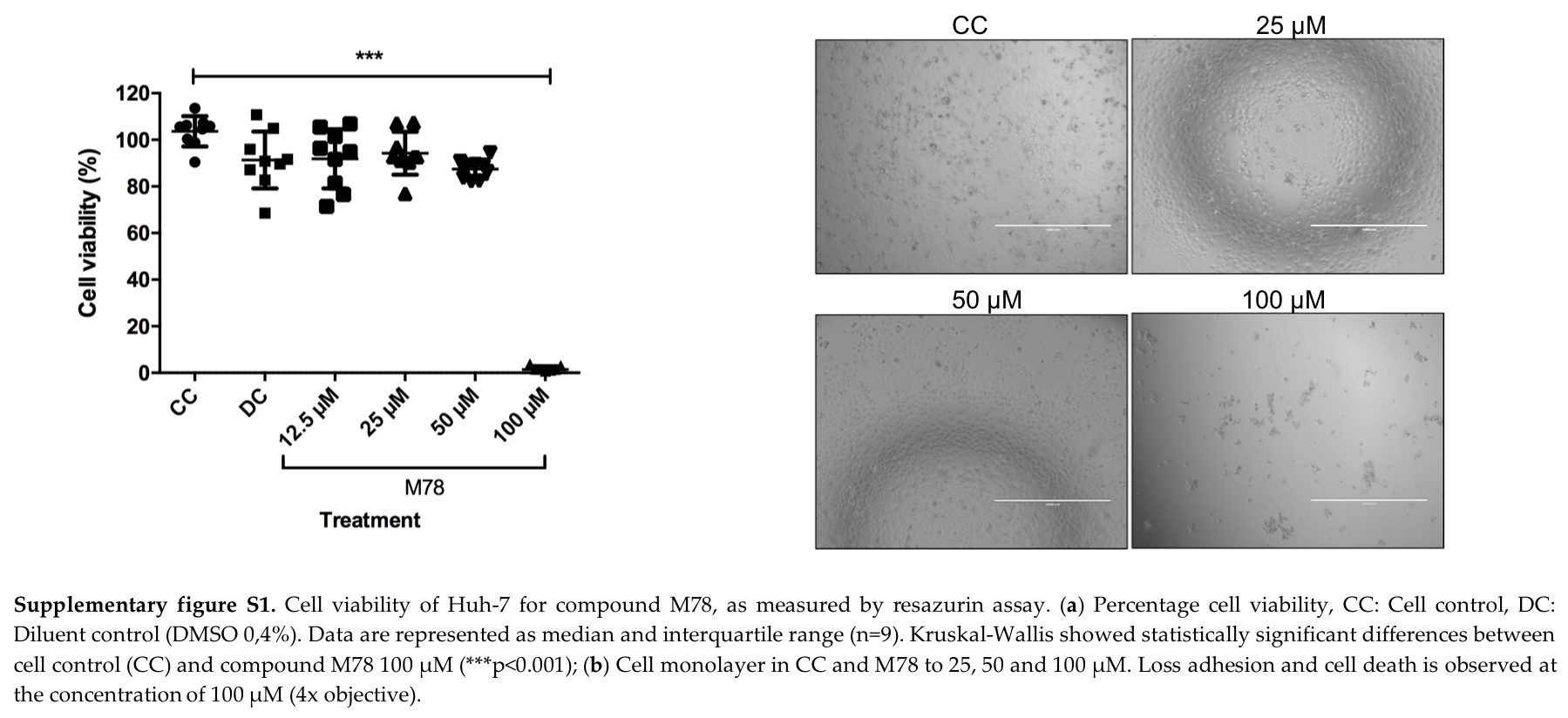

Supplement: Supplementary file 1 [file viruses-15-01563-s001.zip › viruses-2422236-supplementary.png]
